# CXCL1 Clone Evolution Induced by the HDAC Inhibitor Belinostat Might Be a Favorable Prognostic Indicator in Triple-Negative Breast Cancer

**DOI:** 10.1155/2021/5089371

**Published:** 2021-04-17

**Authors:** Xin-le Han, Jun Du, Ya-dan Zheng, Jia-jing Dai, Su-wen Lin, Bing-yue Zhang, Fu-bo Zhong, Zhe-guang Lin, Shu-qi Jiang, Wei Wei, Zheng-yu Fang

**Affiliations:** ^1^Biomedical Research Institute, Shenzhen Peking University-The Hong Kong University of Science and Technology Medical Center, Shenzhen, Guangdong 518036, China; ^2^State Key Laboratory of Experimental Hematology, Institute of Hematology and Blood Disease Hospital, Chinese Academy of Medical Sciences and Peking Union Medical College, Tianjin 300020, China; ^3^Department of Biobank, Peking University Shenzhen Hospital, Shenzhen, Guangdong 518036, China; ^4^Department of Thyroid and Breast Surgery, Peking University Shenzhen Hospital, Shenzhen, Guangdong 518036, China

## Abstract

Triple-negative breast cancer (TNBC) is the most lethal subtype of breast cancer due to its lack of treatment options. Patients with TNBC frequently develop resistance to chemotherapy. As epigenetic-based antineoplastic drugs, histone deacetylase inhibitors (HDACis) have achieved particular efficacy in lymphoma but are less efficacious in solid tumors, and the resistance mechanism remains poorly understood. In this study, the GSE129944 microarray dataset from the Gene Expression Omnibus database was downloaded, and fold changes at the transcriptome level of a TNBC line (MDA-MB-231) after treatment with belinostat were identified. Gene Ontology (GO) and Kyoto Encyclopedia of Genes and Genomes (KEGG) pathway enrichment analyses were used to identify the critical biological processes. Construction and analysis of the protein-protein interaction (PPI) network were performed to screen candidate genes related to cancer prognosis. A total of 465 DEGs were identified, including 240 downregulated and 225 upregulated genes. The cytokine-cytokine receptor pathway was identified as being significantly changed. Furthermore, the expression of CXCL1 was implicated as a favorable factor in the overall survival of breast cancer patients. With *in vitro* approaches, we also showed that belinostat could induce the expression of CXCL1 in another 2 TNBC cell lines (BT-549 and HCC-1937). We speculate that belinostat-induced CXCL1 expression could be one of the results of the stress clone evolution of cells after HDACi treatment. These findings provide new insights into clone evolution during HDACi treatment, which might guide us to a novel perspective that various mutation-targeted treatments should be implemented during the whole treatment cycle.

## 1. Introduction

Histone deacetylases (HDACs) catalyze the N-acetyl group's cleavage from acetylated lysine residues located on the tails of nucleosomal histones. By interacting with histone acetyltransferases (HATs), HDACs regulate histone acetylation and influence the expression level of genes. Besides, many nonhistone protein targets, such as transcription factors, transcription regulators, signal transduction mediators, DNA repair enzymes, nuclear import regulators, chaperone proteins, structural proteins, inflammation mediators, and viral proteins, are also substrates for HDACs. As a result, HDACs control many oncogenes and apoptotic genes' expression levels and hence control many cancer cellular processes, such as proliferation, migration, cell death, and angiogenesis [1–3]. Eighteen mammalian HDAC isoforms have been identified and grouped into four classes based on their phylogenetic homology: class I HDACs (HDACs 1, 2, 3, and 8), class II HDACs (HDACs 4, 5, 6, 7, 9, and 10), class III HDACs (sirtuin family: SIRT1-SIRT7), and class IV HDACs (HDAC 11) [4].

As the first successful application of epigenetic-based cancer therapy, HDAC inhibitors have been discovered to have specific anticancer activities in preclinical studies and clinical treatments [5]. In 2006, suberoylanilide hydroxamic acid (SAHA, vorinostat) was approved by the US FDA to treat cutaneous T-cell lymphoma (CTCL). After that, several HDACis have been approved by the FDA for the treatment of other cancers, including peripheral T-cell lymphoma (PTCL) and multiple myeloma [6, 7]. Until now, epigenetic therapy has achieved specific effects in hematologic neoplasia, which has stimulated growing interest and enthusiasm for further developing epigenetic therapies for other malignancies.

In addition to some progression in T-cell lymphomas and other hematological malignancies, several HDAC inhibitors are efficacious in solid tumors. The results of most clinical trials were in favor of using HDAC inhibitors either before the initiation of chemotherapy or in combination with other treatments [8]. However, their overall effects on chromatin, which can be viewed as positively modulating the expression of many genes, are also likely to generate clinical toxicities, limiting their clinical use. It has been reported that limited penetration and extensive tissue distribution result in clinical ineffectiveness and off-target effects, such as myelosuppression, fatigue, and cardiac toxicity [9]. Hence, the molecular mechanism underlying the inadequate response of HDAC inhibition in solid tumors still needs to be elucidated.

Breast cancer can be divided into 3 subtypes based on the presence or absence of different proteins in breast cancer cells, which have different prognostic and therapeutic implications. Hormone receptor-positive breast cancer accounts for approximately 70% of breast cancer cases and has either estrogen receptor (ER) or progesterone receptor (PR) protein in the cancer cells; human epidermal growth factor receptor 2 (HER2, also known as ERBB2) breast cancer makes up 15% to 20% of breast cancer cases; TNBC is more heterogeneous and lacks ER, PR, and HER2 protein expression, accounting for approximately 15% of all breast cancer cases [10]. Patients with TNBC show poor prognosis and frequently develop resistance to chemotherapy. Since they lack ER, PR, and HER2 receptors, they are not eligible for hormone or anti-HER2 therapy. TNBC patients harbor high levels of somatic mutations, frequent mutations in TP53 (83%), and complex aneuploid rearrangements (80%) that result in extensive intratumor heterogeneity (ITH) [11, 12]. To date, few HDACis have been approved by the US FDA for breast cancer treatment. Notably, chidamide, which selectively targets class I HDACs (subtypes 1, 2, and 3) and class IIb HDACs (subtype 10), has been officially approved by the Chinese National Medical Products Administration (NMPA) for use in combination with aromatase inhibitors in the treatment of locally advanced or metastatic hormone receptor-positive breast cancer that overexpresses HER2 [13]. The benefits from the breakthroughs in HDAC inhibitor research have greatly encouraged the study of their mechanisms in solid tumors.  Table 1 lists several HDAC inhibitors currently being evaluated in a few phase II/III clinical trials. Belinostat is an HDAC inhibitor that has been broadly used to treat PTCL [14]. As a hydroxamic acid-derived pan-HDAC inhibitor that has a high affinity for class I HDACs 1, 2, and 3, class II HDACs 6, 9, and 10, and class IV HDAC 11 [15, 16], belinostat was also evaluated for the treatment of solid cancers, such as lung squamous cell carcinoma [17], renal cancer [18], and hepatocellular carcinoma [19]. Several clinical trials are based on targeting advanced breast cancer, including NCT04315233, NCT03432741, and NCT00413075. The column number of Table 1 should be corrected in sequence.

In recent years, the development of bioinformatics has extensively promoted progress in the field of life sciences. Integrating and reanalyzing the RNA-seq or microarray data using bioinformatics methods may help identify gene regulatory pathways, essential genes, and their associated networks in different diseases, providing information on the possible molecular mechanisms of diseases, potential drug research, and development directions [20, 21]. Some studies have precisely revealed details on tumor microenvironment crosstalk [22, 23] and the effect of epigenetic modification on tumor development [24]. In this study, we found that some cytokines, especially CXCL1, showed a significant difference in expression after treatment with belinostat. Previous literature has reported that CXCL1 is a critical factor in inflammatory diseases and tumor progression. It increases the expression of matrix metalloproteinase (MMP) 2/9 through the ERK1/2 pathway as well as breast cancer metastasis and invasion [25, 26]. This study is aimed at better understanding the potential molecular alterations underlying HDAC inhibitors in breast cancer via bioinformatics methods and *in vitro* experiments, providing a rationale to explore the clinical value of HDACi in solid cancer.

## 2. Materials and Methods

### 2.1. Acquiring RNA Sequencing Profiles and Analysis of Differentially Expressed Genes

Expression profiles from high-throughput sequencing were obtained using the Gene Expression Omnibus (GEO) database. We downloaded the mRNA expression microarray dataset GSE129944, which contained data about MDA-MB-231 cells after treatment with 17-AAG, belinostat, and the combination of 17-AAG with belinostat. Each treatment group was performed in duplicate. The mRNA expression profiles were determined using the Illumina HiSeq 2500 mRNA sequencing platform (Illumina Inc., USA) and normalized as fragments per kilobase of exons per million mapped reads (FPKM) data [27]. Among those sets, we chose the belinostat treatment group to probe the molecular alterations responding to HDAC inhibitors. Gene expression alterations in the treated group were normalized to the corresponding control treated with vehicle. We used the ggplot2 package in R version 4.0 to explore the differentially expressed genes (DEGs) and adopted a threshold cutoff of *p* < 0.05 with absolute log2‐fold change (∣log2 FC∣) ≥ 1.5.

### 2.2. GO and KEGG Enrichment Analyses of Differentially Expressed mRNAs

To understand the functional roles of the differentially expressed mRNAs, DEGs were input into the DAVID (https://david.ncifcrf.gov) [28] and subjected to Gene Ontology (GO) and Kyoto Encyclopedia of Genes and Genomes (KEGG) pathway enrichment analyses [29, 30]. To better understand the differential expression, upregulated and downregulated genes were analyzed separately. Enrichment analysis was carried out to measure the function's significance; the higher the value of enrichment, the more specific the corresponding function, by which the GO term of the associated biological process was identified. KEGG was used to understand the high-level functions and utilities of biological systems as previously mentioned [31]. We used the “topGO” and “pathview” R packages to implement the enrichment analyses. GO terms and KEGG pathways with a corrected *p* < 0.05 were considered significantly enriched.

### 2.3. Protein-Protein Interaction Network

Based on the GO and KEGG pathway analysis, the STRING database (http://string-db.org) was used to construct the protein-protein interaction network of differentially expressed genes [32]. Furthermore, Cytoscape v3.7 was used to identify the core motifs [33]. Through the MCODE function of Cytoscape, modules with the highest score were filtered out. Besides, using the cytoHubba plug-in in Cytoscape v3.7 software, the top 20 nodes were ranked by degree [34].

### 2.4. Hub Gene Screening and Survival Analysis

After acquiring the hub genes, the prognosis was assessed via GEPIA (http://gepia.cancer-pku.cn/) [35]. As the hub genes involved in the GO analysis and pathway analysis were associated with the tumor's biological characteristics, we assessed their prognostic significance. Also, we used the online tool TIMER (https://cistrome.shinyapps.io/timer/) to investigate CXCL1 levels in different cancers [36], and the UALCAN website (http://ualcan.path.uab.edu/) was used to acquire the expression of CXCL1 in BRCA based on breast cancer subtypes [37].

### 2.5. Cell Culture

The cell culture conditions used were as follows: MDA-MB-231 cells were maintained in DMEM with 10% fetal bovine serum (FBS). MDA-MB-435 cells were maintained in Leibovitz's L-15 medium with 10% FBS. BT-549 cells were maintained in RPMI-1640 medium with 0.023 U/ml insulin and 10% FBS. HCC-1937 cells were maintained in RPMI-1640 medium with 10% FBS. MDA-MB-231 and MDA-MB-435 cells were incubated at 37°C without CO_2_. BT-549 and HCC-1937 cells were incubated at 37°C with 5% CO_2_.

### 2.6. Real-Time Quantitative PCR

According to the manufacturer's protocol, total RNA was isolated from cells at the logarithmic phase using the TRIzol reagent (Sigma, USA). First-strand cDNA was synthesized using the GoScript Reverse Transcription System Kit (Promega, USA). Real-time PCR was performed with GoTaq qPCR Master Mix (Promega, USA) using a C1000 Thermal Cycler apparatus (Bio-Rad) in a 20 *μ*l reaction volume to the manufacturer's protocols. The procedure was as follows: 95°C for 3 min, (95°C for 15 s, 60°C for 60 s, and 72°C for 30 s), and 95°C for 10 s, followed by a melt curve analysis (60°C to 95°C, increments of 0.5°C for 20 s) to confirm the specificity of the PCR primers. Ct values for mRNA were normalized to GAPDH. The primers for CXCL1 and GAPDH were as follows: CXCL1 (sense): 5′-TCC AGA GCT TGA AGG TGT TGC C-3′, CXCL1 (antisense): 5′-AAC CAA GGG AGC TTC AGG GTC A-3′, hGAPDH (sense): 5′-CAG CCT CAA GAT CAT CAG CA-3′, and hGAPDH (antisense): 5′-TGT GGT CAT GAG TCC TTC CA-3′. The fold change was calculated using the 2 − ΔΔCt method. Three independent experiments were carried out.

### 2.7. Western Blot Analysis

The cells were lysed in SDS lysis buffer (Beyotime, China) supplemented with a protease inhibitor cocktail (Takara, China). Total protein was quantified by using the BCA Protein Assay Kit (Beyotime, China), and proteins were separated by SDS-PAGE and transferred onto a polyvinylidene fluoride (PVDF) membrane (Pall, USA). The membranes were blocked in BSA (3% *w*/*v* in PBS+0.1% Tween 20) for 30 min at room temperature and incubated with diluted antibodies (Invitrogen, USA) at 4°C overnight. According to the manufacturer's instructions, the proteins were detected by an enhanced chemiluminescence system (Pierce, USA). Three independent experiments were carried out, and the CXCL1 protein data were normalized to *α*-tubulin.

### 2.8. Statistical Analysis

Fold change and Student's *t*-test were employed to evaluate the statistical significance of the results. Differences with *p* < 0.05 between the two groups were considered significant. The *p* value was corrected by calculating the false discovery rate. GO analysis was performed via the DAVID with its statistical tool, the Bonferroni correction method, the Benjamini-Hochberg false discovery rate, and the bootstrap method. KEGG analysis was carried out by Fisher's exact probability test and the gene enrichment analysis included in the DAVID. Pearson correlation coefficients were used to construct the PPI network with Cytoscape software. Then, the gene expression data were clustered hierarchically, and the connection methods used were average linkage and median standardization.

We profiled the general survival information and the transcripts using the univariate Cox proportional hazards regression model. The difference in OS between the high mRNA expression and low mRNA expression groups was assessed by Kaplan-Meier survival curves and the log-rank test. *p* values by two sides less than 0.05 were considered statistically significant. Real-time quantitative PCR and Western blot analysis were performed using one-way ANOVA.

## 3. Results

### 3.1. Data Summary of RNA Sequencing Identification

**Figure 1:**
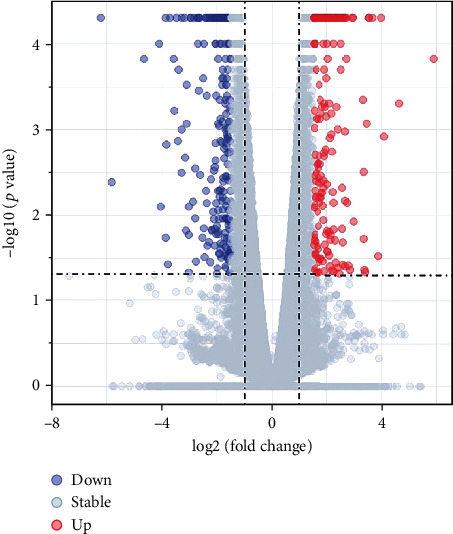
The volcano plot of 465 differentially expressed genes. The *x*-axis represents the mean expression differences of genes between cancer cell lines and normal samples, and the *y*-axis indicates a log-transformed *p* value. ∣log2FC | >1.5 and *p* value < 0.05 were set as the cutoff criteria.

To evaluate the effect of the HDAC inhibitor belinostat on TNBC cells, the GSE129944 dataset and expression profiles from high-throughput sequencing were collected from the GEO database. We used absolute log2 (fold change) ≥ 1.5 and *p* value < 0.05 as cutoff values value. As expected, belinostat treatment led to alterations in the global transcription profile, and 465 DEGs were obtained from pairwise comparisons of samples, including 240 downregulated and 225 upregulated genes. As shown in Figure 1, the DEGs were visualized in the form of a volcano plot.

### 3.2. Belinostat Treatment Results in the Expressional Reprogramming of MDA-MB-231 Cells

**Figure 2:**
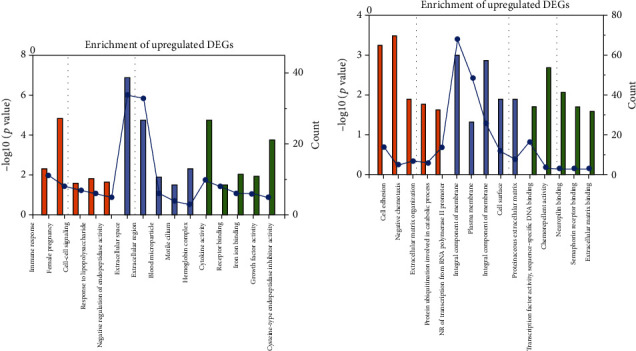
Expressional reprogramming induced by belinostat treatment. Top 15 enriched GO terms for upregulated DEGs (a) and downregulated DEGs (b). The orange, blue, and green colors represent biological processes, cellular components, and molecular functions, respectively.

To further evaluate the transcriptome's functional distribution, the DAVID was used to perform GO and KEGG pathway enrichment analyses of the DEGs [38]. The GO enrichment results showed that the upregulated DEGs' biological processes were mainly involved the immune response, female pregnancy, cell-cell signaling, response to lipopolysaccharide, and negative regulation of endopeptidase activity. The downregulated DEGs were associated with cell adhesion, negative regulation of transcription from the RNA polymerase II promoter, extracellular matrix organization, protein ubiquitination involved in ubiquitin-dependent protein catabolic processes, and negative chemotaxis. Phosphorylation of the RNA polymerase II large subunit is necessary for initiation and elongation of transcription [39]. After the HDACi treatment, the negative regulation of transcription from the RNA polymerase II promoter may lead to cell death in preclinical models of TNBC. Regarding molecular functions, the upregulated DEGs were mainly related to cytokine activity, receptor binding, iron ion binding, growth factor activity, and cysteine-type endopeptidase inhibitor activity. The downregulated DEGs were mainly involved in transcription factor activity, sequence-specific DNA binding, chemorepellent activity, neuropilin binding, semaphorin receptor binding, and extracellular matrix binding. Additionally, the cell component analysis results suggested that the DEGs might be involved in the extracellular region and integral component of the membrane. GO analysis results have some similarities with the previous RNA-seq of TNBC [40], which indicated that the identified genes were associated with the regulation of various biological processes (as shown in Figures 2(a) and 2(b)).

**Figure 3:**
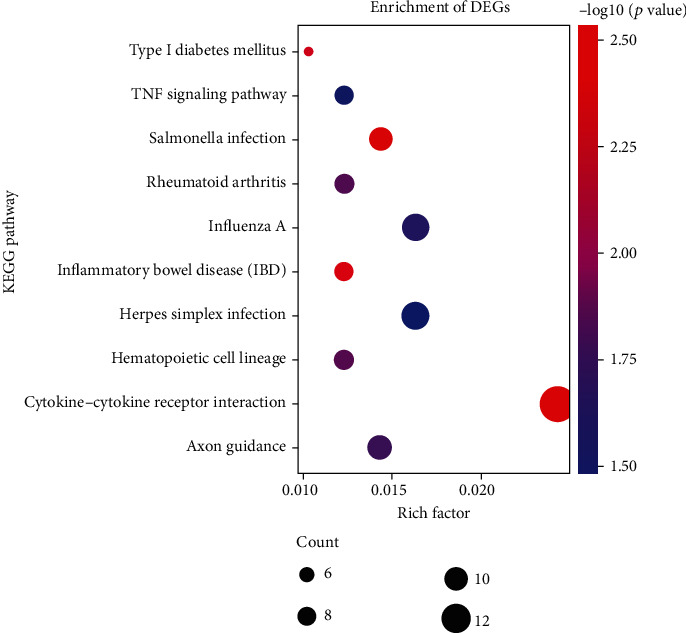
Top 10 enriched KEGG pathways of the differentially expressed mRNAs.

KEGG pathway analysis (Figure 3) demonstrated that the “cytokine-cytokine receptor interaction” was a significant pathway, which corresponded to CXCL1, CSF2, AMH, CXCL3, TNFSF15, IL12A, IL1B, IL24, TNFSF18, and IL1A. Some studies have shown that the cytokine-cytokine receptor interaction pathway plays a critical role in generating an immune-suppressive microenvironment and participates in metastasis and proliferation [34]. Besides, the changes in pathways related to some immune diseases were statistically significant. These annotations provide a valuable resource for investigating biological pathways and gene functions.

### 3.3. PPI Network of DEGs and Screening of Hub Genes

**Figure 4:**
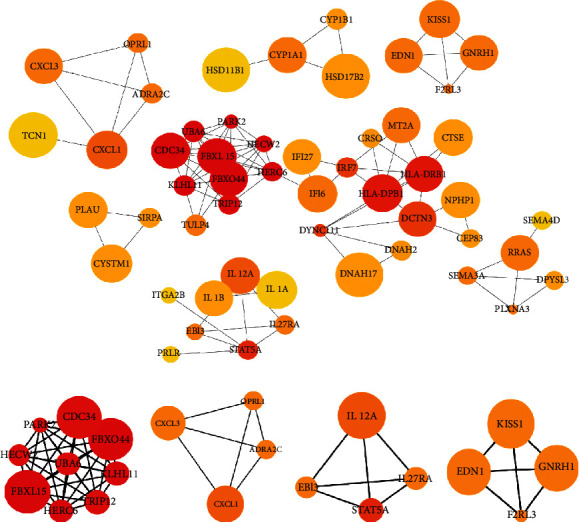
PPI network of DEGs and screening of hub genes. (a) Protein-protein interaction network analysis. Nodes change from red to yellow according to the degree of interaction and become larger as the log2 (fold change) value increases. (b–e) The top four modules with the highest scores.

The exploration and prediction of protein-protein interactions were based on the STRING database (the type of network associations included experiment, coexpression, and database with high confidence interaction scores (0.700)). Cytoscape v3.7 software was then used to construct the protein-protein interaction network of differentially expressed genes (Figure 4(a)). Four modules with the highest scores (9.0, 4.0, 4.0, and 4.0) were filtered out by using MCODE, as shown in Figures 4(b)–4(e). In addition, using the cytoHubba plug-in, the top 20 nodes were ranked by degree, including HERC6, FBXL15, KLHL11, FBXO44, UBA6, PARK2, TRIP12, HECW2, CDC34, HLA-DRB1, HLA-DPB1, STAT5A, IRF7, DYNC1I1, DCTN3, CXCL1, IL12A, CDON, OPRL1, and ADRA2C. The associated signaling cascades include ubiquitination, immune recognition, signal transduction, and transcriptional regulation (https://www.genecards.org/) [41].

### 3.4. Survival Curve and Bioinformatics Prediction

**Figure 5:**
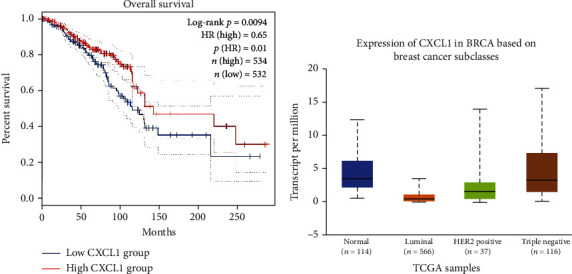
Prognostic value and expression of CXCL1 in breast cancer: (a) Kaplan-Meier survival curves for the association of the secretion of CXCL1 with overall survival; (b) expression of CXCL1 in BRCA based on different subclasses.

**Figure 6:**
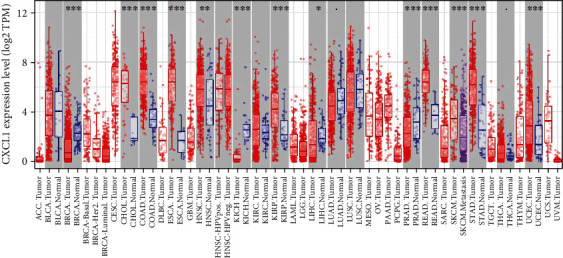
Analysis of CXCL1 across diverse cancer types. ACC: adrenocortical carcinoma; BLCA: bladder urothelial carcinoma; BRCA: breast invasive carcinoma; CESC: cervical squamous cell carcinoma and endocervical adenocarcinoma; CHOL: cholangiocarcinoma; COAD: colon adenocarcinoma; DLBC: lymphoid neoplasm diffuse large B-cell lymphoma; ESCA: esophageal carcinoma; GBM: glioblastoma multiforme; HNSC: head and neck squamous cell carcinoma; KICH: kidney chromophobe; KIRC: kidney renal clear cell carcinoma; KIRP: kidney renal papillary cell carcinoma; LAML: acute myeloid leukemia; LGG: brain lower grade glioma; LIHC: liver hepatocellular carcinoma; LUAD: lung adenocarcinoma; LUSC: lung squamous cell carcinoma; MESO: mesothelioma; OV: ovarian serous cystadenocarcinoma; PAAD: pancreatic adenocarcinoma; PCPG: pheochromocytoma and paraganglioma; PRAD: prostate adenocarcinoma; READ: rectum adenocarcinoma; SARC: sarcoma; SKCM: skin cutaneous melanoma; STAD: stomach adenocarcinoma; TGCT: testicular germ cell tumors; THCA: thyroid carcinoma; THYM: thymoma; UCEC: uterine corpus endometrial carcinoma; UCS: uterine carcinosarcoma; UVM: uveal melanoma.

In this study, the hub genes associated with the tumor's biological characteristics were subjected to survival analysis. Their effects on breast cancer prognosis were assessed via GEPIA (http://gepia.cancer-pku.cn/), a web-based tool developed by Tang that delivers fast and customizable functionalities based on TCGA and GTEx data (Supplementary Table 1). The results showed that the expression of CXCL1 was near related to superior OS in breast cancer patients. The survival curve suggested that OS was significantly shortened in breast cancer patients with low expression of CXCL1 (Figure 5(a)). Furthermore, the UALCAN dataset, which is based on publicly available cancer omics data (TCGA and MET500), was used to estimate the expression of CXCL1 in BRCA based on different subclasses (Figure 5(b) and Supplementary Table 2). The statistical analysis showed that normal vs. TNBC and luminal vs. TNBC had significant differences (*p* < 0.05), but HER2-positive vs. TNBC did not have a noticeable difference. Through the analysis of immune infiltration across diverse cancer types, we found that compared to normal tissue, CXCL1 has low expression in breast cancer, while in cholangiocarcinoma, colon adenocarcinoma, esophageal carcinoma, head and neck squamous cell carcinoma, and hepatocellular liver carcinoma, the situation is the opposite (Figure 6). Commonly, different tumor types or different periods with the same type of tumor may be related to the unique immune microenvironment. CXCL1 has been reported to be associated with metastasis, angiogenesis, and chemoresistance [40, 41]. The objective function of CXCL1 in tumor development and the alteration mechanisms and properties after the HDACi treatment still need to be elaborated.

### 3.5. Belinostat Could Induce the Expression of CXCL1 in Other TNBC Cell Lines

**Figure 7:**
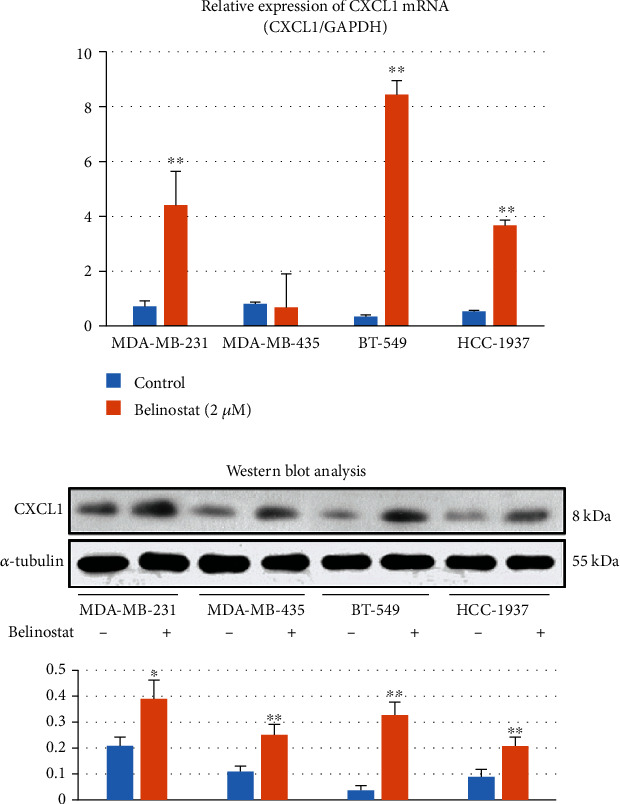
Belinostat induces CXCL1 expression in other TNBC cell lines: (a) real-time PCR shows that belinostat (2 *μ*M) significantly upregulates the mRNA levels of CXCL1 in 4 breast cancer cell lines; (b) Western blot analysis and quantitative analysis show that the addition of belinostat (2 *μ*M) induces the expression of CXCL1 in 4 breast cancer cell lines.

Next, we aimed to investigate whether belinostat treatment could induce the expression of CXCL1 in other breast cancer lines. BT-549, MDA-MB-231, HCC-1937 (TNBC), and MDA-MB-435 (HER2-positive) cell lines were used. As shown in Figure 7(a), after treatment with belinostat for 24 h, a significant increase in CXCL1 mRNA expression was induced in BT-549, MDA-MB-231, and HCC-1937, especially BT-549 cells. The protein levels of CXCL1 were upregulated in BT-549, MDA-MB-435, MDA-MB-231, and HCC-1937 cells after 48 h of belinostat (2 *μ*M) treatment (Figure 7(b)). The changes were statistically significant.

In summary, the induction of CXCL1 expression after belinostat treatment might be an indicator of superior prognosis for TNBC patients, which needs further attention for precision therapy in the setting of this life-threatening disorder.

## 4. Discussion

Although HDAC inhibitors have achieved some success in treating the hematological system's malignant tumors, the results in solid cancers in terms of benefits are less clear. Currently, some clinical trials are underway and tend to combine HDAC inhibitors with chemotherapy or other targeted therapies to enhance clinical efficacy [42, 43]. Generally, HDAC inhibitors lead to the inhibition of tumor growth and apoptosis of cancer cells. A previous study built an acetylation model to predict and verify the cellular metabolic state's impact on sensitivity to drugs that disrupt acetylation and demonstrated the interconnection between metabolism and acetylation [44]. Besides, class II HDAC inhibitors can selectively reprogram monocytes and macrophages in the tumor; reprogramming activates a robust antitumor immune response, mainly mediated by macrophages, CD8+ T-cells, and IFN*γ*, and reduces both primary and metastatic tumor burdens [45]. For example, a selective class IIa histone deacetylase inhibitor, TMP195, influenced human monocyte responses to the colony-stimulating factors CSF-1 and CSF-2 *in vitro* [46]. It alters the tumor microenvironment and reduces tumor burden and metastasis by modulating macrophage phenotypes, including the recruitment and differentiation of highly phagocytic and stimulatory macrophages within tumors [47]. Belinostat broadly inhibits class I HDACs 1, 2, and 3, class II HDACs 6, 9 and 10, and class IV HDAC 11. Indeed, in some human tumors, the overexpression of HDAC6 is associated with more advanced tumor stages and higher tumor invasiveness, so the survival rate is low in cholangiocarcinoma, ovarian cancer, and acute myeloid leukemia (AML) [48–50]. Besides, there might be a connection between the overexpression of HDAC6 in ER-positive cells and ineffective endocrine therapy and poor prognosis [51]. A previous study demonstrated that MDA-MB-231 cells show increased invasiveness and migration compared with MCF-7 cells because they overexpress HDAC6 and matrix metalloproteinase (MMP) 9 [52].

In this study, the cytokine-cytokine receptor pathway, as the top-ranked pathway, stimulated our interest. The DEGs involved in the interaction were transcriptionally modulated, suggesting that the reprogramming of the network in cancer cells was triggered by HDAC inhibition. According to previous reports, CXC chemokines are critical to malignant initiation and cancer progression, in addition to their role in inflammation. Some CXC chemokine family members act as promoters of angiogenesis, including CXCL1, CXCL2, CXCL3, CXCL5, CXCL6, CXCL7, and CXCL8 [53]. Based on the analysis of immune infiltrates across diverse cancer types, abnormal expression of CXCL1 has been found in numerous types of malignancies and has been associated with metastasis, angiogenesis, and chemoresistance [54, 55]. Zeng et al. discovered that the mRNA level alteration of cytokine pathways caused by HDAC led to the downstream response via the LIFR-JAK1-STAT3 signaling-centered feedback loop, which restrained the efficacy of HDAC inhibitors in breast cancer [56].

Notably, the lack of response could be due to drug-induced compensatory alterations arising in both malignant cells and the tumor microenvironment. A previous study reported the evolution of genetic mechanisms of resistance to palbociclib plus fulvestrant in ER-positive breast cancer. It showed that clonal evolution is frequent in response to therapy since acquired driver mutations in growth factor receptors and signal transduction pathways are frequently detected [57]. According to large-scale sequencing results, the level of CXCL1 in normal tissue is higher than that in breast cancer. In our study, CXCL1 was upregulated after treatment with HDACis and predicted a better prognosis, which is consistent with previous results. The CXCL1 mutation induced by belinostat treatment stress might result in DNA damage in tumor cells, which causes the microenvironment to become inadaptive to tumor cell growth. Drug-mediated stress-induced mutagenesis probably acts as a double-edged sword, causing neoplasm necrosis in the context of belinostat intervention [58].

The selective pressure of treatment may lead to intrinsic tumor resistance and the acquisition of an adaptive response. It is not clear whether CXCL1 plays a positive or negative role. In this scenario, defining the various antitumor activities' molecular events is vital for selecting the appropriate HDACi therapy in solid tumors. There are also some limitations in this work. This article was primarily an in silico one. Limited wet lab studies were performed to have the first line of validation of the bioinformatics outputs, but larger sequencing groups of TNBCs need to be analyzed and detailed studies are required for further validation. In the future, we hope to conduct high-throughput sequencing and explore the potential mechanism between HDACis and elevated cytokine levels. In summary, the present findings revealed HDACi treatment-related RNA sequencing alterations in TNBC cells via bioinformatics analyses from the GEO database and *in vitro* experiments. Furthermore, the interaction network of differentially expressed genes revealed that changes in the cytokine-cytokine receptor pathway and CXCL1 secretion might have potential relevance for pharmacogenetic resistance to histone deacetylase inhibitors.

## 5. Conclusions

We downloaded the GSE129944 microarray dataset from the Gene Expression Omnibus database to identify changes at the transcriptome level in MDA-MB-231 breast cancer cells after treatment with belinostat. Unexpectedly, cells that underwent belinostat treatment gained a drug sensitivity advantage to epigenetic medication via CXCL1. That is, breast cancer patients with high expression of CXCL1 have a better prognosis, indicating that CXCL1 could be a novel favorable prognostic indicator. The induction of CXCL1 expression by belinostat treatment was also found in other TNBC cell lines. Detecting the emergence of clonal evolution under the pressure of tumor treatment selection will indicate prognosis and help us understand the disease's development.

## Figures and Tables

**Table 1:** Completed clinical trial of HDAC inhibitors in breast cancer. CR: complete response; PR: partial response; ORR: objective response rate; OS: overall survival; PFS: progression-free survival; AEs: adverse events; TTP: time to disease progression; TTF: time to treatment failure; DoR: duration of response.

No.	NCT number	Interventions	Phases	Enrollment	Status	Outcome
1	NCT00395655	Hydralazine and magnesium valproate	Phase 2	16	Terminated	CR: 31% (5/16); PR: 50% (8/16)
2	NCT01194908	Decitabine, LBH589, and tamoxifen	Phase 1, phase 2	5	Terminated	No study results posted
3	NCT00567879	Panobinostat and Trastuzumab	Phase 1, phase 2	56	Terminated	PR: 1.78% (1/56)
4	NCT00777335	Panobinostat and LBH589	Phase 2	4	Terminated	CR: 0; PR: 0
5	NCT00132002	Vorinostat	Phase 2	14	Terminated	ORR: 0; mean OS: 24 months
6	NCT00262834	Vorinostat	Phase 2	54	Completed	Number of participants with AEs: 17/25
7	NCT01118975	Vorinostat and lapatinib	Phase 1, phase 2	12	Terminated	No study results posted
8	NCT00828854	Entinostat (SNDX-275)	Phase 2	25	Completed	No study results posted
9	NCT00258349	Vorinostat and trastuzumab	Phase 1, phase 2	16	Completed	CR: 0; PR: 0; mean TTP: 1.5 months. Mean OS: 9.3 months
10	NCT00365599	Vorinostat and tamoxifen	Phase 2	43	Completed	ORR: 18.6% (8/43); mean TTP: 10.3 months. Number of participants with serious AEs: 4/43
11	NCT00676663	Entinostat and exemestane	Phase 2	64	Completed	ORR: 4.7%
12	NCT01194427	Vorinostat and tamoxifen	Phase 2	2	Terminated	No study results posted
13	NCT00126451	MK0683, vorinostat, and suberoylanilide hydroxamic acid (SAHA)	Phase 2	16	Terminated	TTP: 33.5 days. Number of participants with serious AEs: 11/16
14	NCT01105312	Letrozole and panobinostat	Phase 1, phase 2	28	Completed	CR: 0; PR: 0; mean survival time: 16.1 months; mean TTP: 2.1 months; PFS: 2.1 months; TTF: 2.1 months
15	NCT00368875	Vorinostat, paclitaxel, and bevacizumab	Phase 1, phase 2	53	Completed	CR: 4% (2/53); PR: 45% (24/53); mean PFS: 11.9 months; mean OS: 29.4 months; TTF: 0
16	NCT01234532	Entinostat and anastrozole	Phase 2	5	Terminated	No study results posted
17	NCT00574587	Vorinostat, paclitaxel, trastuzumab, doxorubicin, and cyclophosphamide	Phase 1, phase 2	55	Completed	CR: 33.3% (17/51)
18	NCT00777049	Panobinostat	Phase 2	54	Completed	CR: (1.9%) 1/54; PR: (1.9%) 1/54
19	NCT02395627	Tamoxifen, vorinostat, and pembrolizumab	Phase 2	38	Terminated	ORR: 6.67%; DoR: 17.0 months (group A), 8.8 months (group B); mean PFS: 2.57 months (group A), 2.63 months (group B); mean OS: 14.3 months (group A), 15.0 months (group B), and 7.8 months (group C)

## Data Availability

The datasets used and analyzed during the present study are available from the GSE129944 (https://www.ncbi.nlm.nih.gov/geo/query/acc.cgi?acc=GSE129944).

## Supplementary Materials

Supplementary Materials Supplementary Table 1: the survival analyses of the top 20 nodes ranked by cytoHubba. Supplementary Table 2: comparison expression of CXCL1 in BRCA based on subclasses.
