# Supplementary material for: CXCL1 Clone Evolution Induced by the HDAC Inhibitor Belinostat Might Be a Favorable Prognostic Indicator in Triple-Negative Breast Cancer
Source: Biomed Res Int. 2021 Apr 17;2021:5089371. doi: 10.1155/2021/5089371 (PMC8075662; doi:10.1155/2021/5089371)
Supplement: Supplementary Materials — Supplementary Table 1: the survival analyses of the top 20 nodes ranked by cytoHubba. Supplementary Table 2: comparison expression of CXCL1 in BRCA based on subclasses. [file 5089371.f1.docx]

**Supplementary Data**

**Supplementary Table 1.** The survival analyses of the top 20 nodes ranked by Cytohubba.

| **Gene** | **OS (p-value)** | **HR** | **HR (p-value)** |
| --- | --- | --- | --- |
| HERC6 | 0.8 | 1 | 0.79 |
| FBXL15 | 0.21 | 1.2 | 0.21 |
| KLHL11 | 0.56 | 0.1 | 0.57 |
| FBXO44 | 0.64 | 1.1 | 0.64 |
| UBA6 | 0.39 | 1.2 | 0.39 |
| PARK2 | 0.64 | 1.1 | 0.64 |
| TRIP12 | 0.18 | 1.2 | 0.18 |
| HECW2 | 0.91 | 0.98 | 0.91 |
| CDC34 | 0.86 | 1 | 0.85 |
| HLA-DRB1 | 0.053 | 0.73 | 0.055 |
| HLA-DPB1 | 0.083 | 0.75 | 0.085 |
| STAT5A | 0.32 | 0.85 | 0.33 |
| IRF7 | 0.11 | 0.77 | 0.11 |
| DYNC1I1 | 0.93 | 1 | 0.93 |
| DCTN3 | 0.26 | 1.2 | 0.26 |
| **CXCL1** | **0.0094** | **0.65** | **0.01** |
| IL12A | 0.98 | 0.99 | 0.97 |
| CDON | 0.61 | 0.92 | 0.61 |
| OPRL1 | 0.4 | 0.87 | 0.41 |
| ADRA2C | 0.11 | 1.3 | 0.11 |

**Supplementary Table 2.** Comparison expression of CXCL1 in BRCA based on subclasses.

| **Comparison** | **Statistical significance** |
| --- | --- |
| Normal-vs-Luminal | 5.2234000003093E-07 |
| Normal-vs-HER2 Positive | 2.901800E-01 |
| Normal-vs-TNBC | 3.415200E-03 |
| Luminal-vs-HER2 Positive | 4.545800E-02 |
| Luminal-vs-TNBC | 8.10770000000405E-05 |
| HER2 Positive-vs-TNBC | 2.124800E-01 |
